# Removing ocular artifacts from magnetoencephalographic data on naturalistic reading of continuous texts

**DOI:** 10.3389/fnins.2022.974162

**Published:** 2022-12-22

**Authors:** Sasu Mäkelä, Jan Kujala, Riitta Salmelin

**Affiliations:** ^1^Department of Neuroscience and Biomedical Engineering, School of Science, Aalto University, Espoo, Finland; ^2^Aalto NeuroImaging, Aalto University, Espoo, Finland; ^3^Department of Psychology, University of Jyväskylä, Jyväskylä, Finland

**Keywords:** naturalistic task, eye movement, electrophysiological recording, electromagnetic brain mapping, independent component analysis, reading, language

## Abstract

Naturalistic reading paradigms and stimuli consisting of long continuous texts are essential for characterizing the cortical basis of reading. Due to the highly dynamic nature of the reading process, electrophysiological brain imaging methods with high spatial and temporal resolution, such as magnetoencephalography (MEG), are ideal for tracking them. However, as electrophysiological recordings are sensitive to electromagnetic artifacts, data recorded during naturalistic reading is confounded by ocular artifacts. In this study, we evaluate two different pipelines for removing ocular artifacts from MEG data collected during continuous, naturalistic reading, with the focus on saccades and blinks. Both pipeline alternatives are based on blind source separation methods but differ fundamentally in their approach. The first alternative is a multi-part process, in which saccades are first extracted by applying Second-Order Blind Identification (SOBI) and, subsequently, FastICA is used to extract blinks. The other alternative uses a single powerful method, Adaptive Mixture ICA (AMICA), to remove all artifact types at once. The pipelines were tested, and their effects compared on MEG data recorded from 13 subjects in a naturalistic reading task where the subjects read texts with the length of multiple pages. Both pipelines performed well, extracting the artifacts in a single component per artifact type in most subjects. Signal power was reduced across the whole cortex in all studied frequency bands from 1 to 90 Hz, but especially in the frontal cortex and temporal pole. The results were largely similar for the two pipelines, with the exception that SOBI-FastICA reduced signal in the right frontal cortex in all studied frequency bands more than AMICA. However, there was considerable interindividual variation in the effects of the pipelines. As a holistic conclusion, we choose to recommend AMICA for removing artifacts from MEG data on naturalistic reading but note that the SOBI-FastICA pipeline has also various favorable characteristics.

## 1 Introduction

Reading is one of the most important forms of human communication. A thorough understanding of its neural basis cannot be achieved by using very tightly controlled stimuli, such as isolated words, and constraining eye movements. Instead, it requires the use of some forms of naturalistic paradigms, where the test subjects read continuous text passages, moving their eyes freely. Thus far, the length of the stimulus texts in the majority of neuroimaging studies on naturalistic reading has been limited to the level of single sentences (e.g., Dimigen et al., 2011; Kretzschmar et al., 2015; Metzner et al., 2015, 2017; Vignali et al., 2016; Pfeiffer et al., 2020). In order to study how the reading process unwinds over longer stretches of continuous text, studies with stimuli consisting of several sentences, lines or even pages are required.

The study of the naturalistic reading process greatly benefits from using an imaging method that affords dynamic tracking of brain activity with combined high temporal and spatial resolution, such as magnetoencephalography (MEG). This poses a critical challenge, since electrophysiological methods are sensitive to various electromagnetic artifacts. As eye movements are an integral part of naturalistic reading (Rayner, 1998; Dimigen et al., 2011; Dambacher et al., 2012; Schotter et al., 2014; Kornrumpf et al., 2016; Metzner et al., 2017), ocular artifacts are especially problematic in reading studies.

Eye movements lead to disturbances in the measured signals as a result of several current sources forming a corneo-retinal potential, which can be approximated as a single dipole with a positive pole on the corneal end and a negative pole on the retinal end (Lins et al., 1993). This dipole is approximately parallel to the axis of the eye, and therefore closely follows the direction of the gaze. The most notable type of eye movements are the saccades, which shift the gaze from one location of interest to another. In the case of reading, saccades move the gaze mainly horizontally along the text. During fixations other types of eye movements occur, such as microsaccades, tremor, and drift. However, the size of these movements and the size of the artifacts caused by them is much smaller than that of the saccades (Rolfs, 2009). Furthermore, since the duration of fixations in reading is typically relatively short (Rayner, 2009), the effects of tremor and drift are diminished even further and the temporal intervals become very narrow for microsaccades to occur [general occurrence rate of microsaccades is ca. 1–2 per second (Martinez-Conde et al., 2004; Rucci and Poletti, 2015)]. Blinks, in turn, cause measurement artifacts unrelated to the direction of the gaze. They appear in the measurements as solitary spikes which result mainly from the eyelid sliding over the eyeball and short-circuiting the ocular dipole (Barry and Jones, 1965; Matsuo et al., 1975; Antervo et al., 1985; Lins et al., 1993). Additionally, saccadic spike fields manifest as low-amplitude spikes appearing immediately before saccades and often overlapping with the saccades. Saccadic spike fields originate at the extraocular muscles which rotate the eyeball during a saccade (see e.g., Carl et al., 2012). Saccadic spike fields are particularly problematic when the focus is on induced event-related activity and the task causes very minor eye movements to occur at a task-dependent rate (Yuval-Greenberg et al., 2008). However, in studies concentrating on continuous data instead of isolated events, the effect of saccadic spike fields on analysis results is minimal due to their small size.

Accordingly, saccades and blinks are presumably the key ocular artifacts that need to be explicitly removed from MEG data collected during continuous reading. While the literature on the exact nature of saccade and blink artifacts in MEG recordings is very limited, they have been studied extensively with EEG, and the general EEG observations apply also to MEG. In EEG, the saccade artifact appears as a change of the signal offset, with the size depending roughly linearly on the size of the saccade (Lins et al., 1993; Plöchl et al., 2012). Since the saccade artifact originates at the eyeball, it affects mostly the frontal and fronto-temporal field patterns. The dipolar origin of the saccade signal leads to an increase of amplitude in the electrodes on the same side with the target of the saccade, whereas the electrodes on the opposite side show a decrease (Lins et al., 1993; Plöchl et al., 2012). Spectrally, saccades lie mainly in the frequency range of 4–20 Hz (Keren et al., 2010). The way eyes are moved while reading varies greatly between readers and according to text properties (Rayner, 2009). However, as each reader moves her/his eyes in an individually characteristic pattern, the within-subject variance in saccade length and fixation duration is considerably smaller. As a result, when the saccade artifacts are observed as a continuous signal, the signal is moderately periodic. During reading most saccades are targeted so that they move the gaze forward in the text in the direction of reading. Regressions, which are saccades that move the gaze backward in the text, constitute 10–15% of saccades for skilled readers (Rayner, 2009). Return sweeps are long saccades that occur at the end of one line and take the gaze to the beginning of the next one.

Blinks appear in MEG recordings as sharp peaks lasting usually for some hundreds of milliseconds. The effect of blinks is strongest on the most frontal sensors bilaterally. Contrary to saccades, the spatial distribution of blinks does not vary between different types of blinks. The power spectrum of blinks is mostly concentrated below 5 Hz (Keren et al., 2010).

The saccade and blink artifacts overlap with the cerebral activity of interest both temporally and spectrally. This is especially true for saccades which, during continuous reading, occur at a high rate and cover a wide spectral range. Thus, the effect of the artifacts cannot be avoided by concentrating solely on artifact-free time periods nor by attenuating activity within specific frequency bands. Since these simple techniques are insufficient for coping with ocular artifacts, more sophisticated methods are required.

One possible method is linear regression. It is based on selecting one or several reference channels which accurately represent the artifacts and forming a weighted combination of these channels that is subtracted from the measured data. In the case of ocular artifacts, the reference channels are usually formed by a combination of electro-oculographic signals (EOG) recorded horizontally and vertically. The often-noted drawback of regression methods is that EOG recordings inevitably contain also frontal cerebral activity, which will be erased from the recordings along with the ocular activity. In the context of an MEG study, using regression methods with EOG as a reference is problematic also because EOG measures variations in electric field, whereas MEG measures variations in magnetic field. Thus, although the ocular signals picked up by EOG and MEG are generated by the same processes, they cannot be assumed to be identical enough for a direct subtraction. This problem is partially fixed in adaptive filtering (He et al., 2007). However, even though adaptive filtering offers an improvement to linear regression methods, it is not completely free of the general problems stemming from the use of EOG as a reference and is also confounded by the spectral overlap of ocular artifacts and cortical activity (He et al., 2007).

Another alternative for removing ocular artifacts is to use wavelet transform either as the only method (e.g., Zikov et al., 2002; Krishnaveni et al., 2006a,b) or as an additional processing step in combination with other methods (e.g., Castellanos and Makarov, 2006; Akhtar et al., 2012; Mammone et al., 2012; Mowla et al., 2015). However, although the wavelet transform does not rely on sinusoids and thus its signal decomposition capabilities are not explicitly tied to the frequency spectra of the analyzed signal, it has still been observed to have problems in differentiating ocular artifacts from cerebral activity (Krishnaveni et al., 2006a).

Currently, perhaps the most popular family of methods in artifact removal are blind source separation (BSS) methods. BSS methods (Jutten and Herault, 1991) are based on the assumption that the processed data is a mixture of individual signals, and the goal of BSS methods is to unmix the data into these original components. In artifact removal, these methods are used by first performing the unmixing and then identifying and removing the signals corresponding to artifacts. Among the most notable subfamilies of BSS methods are methods that unmix the signals based on features of their temporal structure, and independent component analysis (ICA) methods, which assume the component signals to be statistically independent.

In the context of ocular artifact removal, BSS methods have several advantages. Firstly, in the unmixing process, each separated signal receives a factor matrix, in which each MEG sensor is assigned a weight corresponding to the strength of the signal on that sensor, essentially enabling BSS methods to take advantage of the characteristic sensor topographies of different ocular artifacts. Secondly, BSS methods can separate signals with similarities in their power spectra, meaning that they are not restricted by an overlap in the spectra of ocular artifacts and cerebral signals. Thirdly, BSS methods can perform the unmixing without the aid of any external reference signal which may not completely correspond to the artifacts. Due to these factors, we consider BSS methods to be inherently superior to the previously introduced alternatives in ocular artifact removal.

In continuous reading, saccades are roughly periodically occurring minor changes of amplitude whereas blinks appear sporadically as prominent spikes. These artifacts are thus characterized by very different traits, and it is questionable whether both types of artifacts can be efficiently extracted with just a single method. In this study, we address this question, with the aim to formulate an efficient scheme for erasing saccades and blinks, by evaluating two alternative ways for ocular artifact removal: (i) a two-stage pipeline where saccades and blinks are removed consecutively by two different signal processing methods that are tuned to the characteristic features of each artifact type, and (ii) a one-stage pipeline where the artifacts are removed simultaneously using a single purportedly powerful method. In the two-stage approach, we remove the saccades using Second-Order Blind Identification (SOBI), a method that is suitable for the task as it segregates the measured data into separate component signals based on differences in their temporally periodic structures. Our choice for removing blinks is FastICA, a method that separates the measured data into statistically independent components by maximizing their negentropy, which we believe to be a suitable objective for identifying the blinks that appear in the MEG recordings as highly random events. As the one-stage method we apply Adaptive Mixture ICA (AMICA) which decomposes the data into independent components by estimating their probability density functions with generalized Gaussian distributions. AMICA has proven promising in a number of tests (e.g., Delorme et al., 2012) and was chosen because of its good test performance and the seeming versatility of its operating principle in estimating different types of components.

The methods are tested using MEG data from a naturalistic continuous reading experiment where the participants read various text passages with the length of multiple pages. The experiment also included a scanning task with similar stimuli as a control condition. Data from both the reading and control tasks were used in the artifact removal process, but the results of the method comparison are derived solely from the reading data.

## 2 Materials and methods

The following sections describe the data set that was used for testing the artifact removal pipelines, the details of the applied methods, the approach for identifying the blink and saccade components, and the methodology for evaluating the effects of the artifact removal.

### 2.1 Data

The data used in this study was originally collected as a part of a naturalistic continuous reading experiment designed to investigate reading-related brain activity (unpublished data) but is used here for examining reading-related ocular artifacts due to the data’s suitability for this purpose. In the experiment, the subjects read connected text, instructed to do so in their own usual way. The stimuli were extracted from various novels or essays in Finnish and used in their original or slightly edited form. The texts were presented as excerpts of three pages, with each page consisting of eight lines of black text on a white background, projected on a screen approximately 1 m in front of the participant. The visual angle formed by each page full of text was 15.4° horizontally and 7.7° vertically. The experiment also included a scanning task in which the subjects searched for inverted letters from texts similar to the ones used in the reading task. The subjects were instructed to move their eyes during this task as if they were reading. The scanning task data was used in the BSS component estimation, but the final effect of artifact removal was evaluated only on the reading task data. All data of each subject were measured in a single session.

The data of 13 right-handed Finnish-speaking subjects (7 male, 6 female, age 20–50 years, mean 25.4 years) with normal or corrected-to-normal vision was used in the study. None of the subjects reported a history of neurological abnormalities or psychiatric disorders. Informed consent was obtained from all subjects, in agreement with the prior approval of the Helsinki and Uusimaa Ethics Committee. The subjects’ cortical activity was measured using a 306-channel MEGIN Vectorview device (MEGIN Oy; Helsinki, Finland) in the Aalto University MEG Core. The device records electromagnetic field at 102 locations, with two planar gradiometers and one magnetometer at each site; only the data recorded by the gradiometers was used in this study. The recordings were bandpass filtered at 0.03–200 Hz and sampled at 600 Hz. The signals were further preprocessed with the MEGIN MaxFilter 2.2 software (MEGIN Oy; Helsinki, Finland) to remove the effect of external magnetic fields on the data via spatiotemporal signal space separation (Taulu and Simola, 2006). EOG was recorded with electrodes placed on the left side of the left eye and on the right side of the right eye (horizontal EOG), and above and below of the left eye (vertical EOG). Eye movements were further monitored with an Eye Link 1000 eye tracker (SR Research Ltd.; Mississauga, ON, Canada) using a sampling rate of 1,000 Hz. Structural magnetic resonance images (MRI) of the subjects were obtained in the Aalto University Advanced Magnetic Imaging Centre with a 3 T Signa EXCITE scanner (GE Healthcare; Helsinki, Finland). The MEG and MRI coordinate systems were aligned with the help of head position coils and their measured locations with respect to anatomical landmarks.

The amount of reading task data used in this study varied between 8.6 and 18.1 min per subject (mean 11.8 min). In the scanning task the amount of data varied from 5.7 to 19.2 min per subject (mean 10.2 min). When feeding the data to the artifact removal pipelines, the data corresponding to each read page were concatenated to form a single continuous recording. Before the concatenation, each channel in the single page units was normalized to have zero mean. Data from the scanning task was also included in the concatenated recording. After the BSS estimations, the scanning data was discarded from the rest of the study and the results were derived using only the reading data. Since FastICA and AMICA cannot directly process numbers which are as small as those normally in MEG measurements, the data was multiplied by a factor of 1e10 for FastICA and 1e8 for AMICA when fed to the methods; after the components had been estimated, they were rescaled to the same scale as the original data.

For one subject FastICA did not extract blinks successfully when using the normal data input scheme. This problem was a result of the subject making only very few blinks. To increase their concentration in the data, only one-page units containing blinks (based on eye tracker data) were included in the data which was fed to FastICA. After estimating the ICA decomposition with this limited data, the estimated demixing matrix was used to calculate the ICA components for all page units. This differing data input scheme was not needed with AMICA.

### 2.2 Blind source separation

BSS methods are based on the assumption that the observed signals *x*(*t*) are a linear mixture of unobserved source signals *s*(*t*)


(1) x⁢(t)=As⁢(t)+n⁢(t),

where *x*(*t*) is an *m*-dimensional random variable denoting an observed signal at time *t*, *A* the linear mixing matrix, size *m*x*n*, *s*(*t*) the values of the *n* source signals at time *t* and *n*(*t*) a noise or a bias term. Some methods omit *n*(*t*). Since the mixing is linear, the original source signals can be obtained by determining an *n*x*m* demixing matrix *W* [and omitting *n*(*t*)], yielding the transformation


(2) s⁢(t)=Wx⁢(t)

*A*, *s*(*t*), and *W* are all unknown and the goal of the BSS methods is to obtain *s*(*t*) by determining *W*. *W* cannot be calculated directly and is therefore estimated such that it yields a demixing that possesses some desired property which differs from method to method. Most methods, like SOBI and FastICA, assume that the observed signals *x*(*t*) are statistically stationary processes. However, some methods, such as AMICA, do not make this assumption.

### 2.3 Second-Order Blind Identification

Second-Order Blind Identification (SOBI) approaches the BSS problem by decorrelating the observed signals *x*(*t*) at various time lags, which is done by diagonalizing a set of covariance matrices estimated from the data with various time differences. Since the different covariance matrices effectively capture the temporal dynamics of cross-dependencies between the observed signals, this approach makes SOBI especially useful for extracting source signals with a distinct temporal structure. The first version of the method was presented by Belouchrani et al. (1997). We used a version of the method written by Belouchrani and Cichocki (2000) and modified by Delorme et al. (2007). This version does not have a version number, but can be found as a part of both EEGLAB (Delorme and Makeig, 2004) and FieldTrip (Oostenveld et al., 2011) toolboxes and from various codesharing services^1^ (Oostenveld, 2010).

The key parameter in the use of SOBI is the set of time lags to be used, since they determine the covariance matrices to be diagonalized and thus the only data that the method truly uses to solve the BSS problem. Since we were foremost interested in using SOBI to extract saccades, we utilized EOG in selecting the time lags. We calculated the autocorrelation of the horizontal EOG recordings to see how the periodicity of saccades manifests with different delays. Most saccades during reading are directed sideways, and the corresponding artifacts thus appear most saliently in the horizontal EOG. This analysis showed that the autocorrelation decreases steadily as the lag increases but in some subjects it may increase again at very high lags, presumably as the result of moving the gaze from one row to the next. Consequently, we chose all time lags from the interval of {0,1,2…,150} corresponding to the delays from 0 to 150 samples and additionally time lags for which the horizontal EOG of the studied individual had an autocorrelation of 0.3 or higher. In order to enable this completely free selection of time lags instead of using all time lags up to a given limit, we also made a very minor edit to the code of the SOBI implementation we used. A more detailed introduction to the basics of the SOBI algorithm is given in the Supplementary Appendix.

### 2.4 FastICA

Independent component analysis (Jutten and Herault, 1991; Comon, 1994) methods strive to solve the BSS problem by seeking a transformation of the data which yields maximally independent components in a statistical sense. This approach is well motivated, since usually the original signals are more independent than their mixtures. However, the independence of the signals cannot be determined without knowing the probability distributions that generated them. Therefore, the version of FastICA used in this study [FastICA for Matlab 2.5^2^ (Karhunen, 2013)] and introduced by Hyvärinen (1999) strives to obtain an independence-maximizing transformation by seeking one that minimizes the mutual information of the components. The mutual information is expressed in an alternative form based on negentropy. The basics of FastICA are introduced in the Supplementary Appendix along with our choices for the contrast function and orthogonalization parameters.

### 2.5 AMICA

AMICA is another ICA method, proposed by Palmer et al. (2008). One key difference between AMICA and the majority of BSS methods is that AMICA does not assume the observed signals *x*(*t*) to be stationary, which leads to the possibility of different models being valid at different times. Consequently, AMICA offers the possibility of estimating several models of the same data simultaneously. This is a welcome option in artifact removal, not because we would expect the distributions of the artifact signals to alter drastically during the measurements, but because the separate models can be estimated by concentrating on different features of the data, thereby potentially uncovering multiple aspects of the artifact signals and increasing the possibility of extracting them more thoroughly. Like FastICA, AMICA approaches the BSS problem by seeking maximally independent components *s*(*t*), but it does it by directly estimating the components’ probability distributions which are approximated as a mixture of several generalized Gaussian distributions or Gaussian scale mixtures. This approach brings flexibility to the component estimation by allowing their probability distributions to be composed of numerous co-distributions, which themselves are also more flexible than many other probability distributions used in the context of BSS. In contrast, for example FastICA assumes the components *s*(*t*) to have a probability distribution of a quite restricted and well-defined form. A mathematically more comprehensive introduction to AMICA is given in the Supplementary Appendix.

The software implementation of AMICA used in this study [AMICA 1.5^3^ (Palmer, 2015)] has a wide range of options that can be altered. For almost all of the options we used their default values, including the number of mixtures to be used for each component, which we kept at three. The only exception was the number of models to be estimated. The default value is one model, which did not yield satisfying results in a few cases. For these subjects we increased the number of models to three, which resulted in sufficient results every time. Having three as a default number of models would also be possible, but since estimating three models and inspecting components from three models is more time-consuming than with one model, we used three models only when necessary. Estimating several models may lead to situations where the saccades are extracted the best in one model, but the clearest blink component is found in another model. As different models have different mixing matrices, the components are not directly mixable between models. In these cases, we removed the targeted artifact components in one model, mixed the components back to the original signal space with the model’s own mixing matrix, then demixed the data with the other model’s demixing matrix to obtain that model’s components and finally removed the artifact components which were estimated the best in that model.

The maximum number of iterations allowed for estimating the models is one of the several adjustable parameters offered in AMICA, and as with most other parameters, we kept its value at the default, which is 2,000 iterations. In our tests, this limit systematically acted as the main stopping criterion for AMICA. We examined how many iterations it would take until AMICA would stop as a result of reaching its other stopping criterion, the minimum learning rate (default 1e-8), which essentially measures the convergence of the whole model; in these cases, AMICA ran for well over 10,000 iterations even exceeding 20,000 iterations for some subjects. However, the main ocular artifact components estimated using these two limiting criteria had Pearson correlations between time series almost systematically c. 0.999, indicating very high similarity. Ocular artifact components thus seem to converge well already in 2,000 iterations.

### 2.6 Component selection

The components corresponding to artifacts were primarily selected by inspecting the components’ time series visually and searching for the ones that express the signal shapes characteristic to saccade and blink artifacts. As an additional verification the sensor topographies of the components were also inspected visually to see whether the strongest weights lie on sensors the artifacts are known to affect the most. The sensor topography information was exploited even further by calculating rough source localization estimates for the components. This was done by first creating a grid with 7-mm spacing covering each subject’s brain and calculating the leadfields in every grid point. Subsequently, correlations between the leadfields and components’ sensor weight matrices were calculated and sorted from highest to lowest for every component. Finally, 500 highest correlations were selected and the grid points corresponding to them were plotted to see which gridpoints had a leadfield best corresponding to the component’s sensor topography and thus were most likely to be the source of the component. For ocular artifacts, the expected grid point spread was such that most of the 500 highest correlated points were in the eye area, its immediate vicinity, or at the edges of the head near the eye area. The analyses required for component selection were conducted by using a combination of various in-house-developed Matlab codes and MEGIN Graph 2.94.6 and MEGIN Mrilab 1.7.25 softwares (MEGIN Oy; Helsinki, Finland).

### 2.7 Comparing the artifact removal methods

The performance of the two different artifact removal pipelines was evaluated in two parts. In the first part, we examined the components which were extracted by the methods and labeled as artifacts. First, we listed the number of saccade and blink components per pipeline and per subject. Then, we quantified the similarity of the components extracted by the two pipelines by calculating the Pearson correlations between the sensor topographies of the components, for each subject. If a pipeline yielded several components for the same artifact, the analysis was conducted only by using the components which were most purely artifactual (denoted as “primary components” from here on).

In the second part, we compared the effects of removing the artifact components from the MEG data. We estimated the spatial distribution of signal power on selected frequency bands using Dynamic Imaging of Coherent Sources (DICS) (Gross et al., 2001). Spatial distributions of signal power were estimated separately for the original (uncleaned) data and data processed with each of the two artifact removal pipelines. The differences between these distributions (SOBI-FastICA vs. uncleaned, AMICA vs. uncleaned, SOBI-FastICA vs. AMICA) were calculated to visualize the effects of the pipelines. For the DICS calculations, one subject’s brain was first fitted with a surface-based grid (9-mm spacing along the surface of the cortex) that was transformed into the other subjects’ brains where the calculations were performed. This procedure yields spatially equivalent grid points across the subjects and thus facilitates robust group-level analysis. To ensure comparability, all DICS calculations were performed with the same DICS filter for which weights were calculated by using a cross-spectral density matrix averaged across the cross-spectral density matrices of data cleaned with the two different pipelines and the uncleaned data. Here, only the number of singular values within the lowest ranked cleaned data was used in the estimation of the pseudoinverse of the cross spectral density matrix and the determination of the beamformer weights, whereas these weights were then applied to the condition specific fully ranked CSD for estimating the cortical level signal power. We normalized all calculated power differences by dividing the difference in each point by either the original signal power in the same point in the uncleaned data (cleaned vs. uncleaned) or the power reduced by using AMICA (SOBI-FastICA vs. AMICA). Comparisons were done both on group and individual subject levels and for six different frequency bands: 1–4, 5–8, 8–13, 15–25, 31–47, and 60–90 Hz.

We examined the subject-level variation in the effects of the pipelines further by selecting all combinations of parcels [according to Desikan-Killiany atlas (Desikan et al., 2006)] and frequency bands which showed the largest group-level differences in power reduction between the two pipelines. Finally, systematic group-level differences in the effects of the pipelines were evaluated by conducting two-sided *t*-tests (*p* < 0.005, uncorrected) on the signal reduction differences between the pipelines over all points, separately for each frequency band. Additionally, the group-level mean differences were examined for all parcel-frequency band combinations which contained points with systematic group-level differences detected in the *t*-tests. Our aim was to identify possible large differences which appear consistently from subject to subject, instead of focusing on statistically significant differences; this is why we chose not to correct for multiple comparisons. The comparison of the artifact removal methods was conducted using in-house-developed Matlab code. The visualizations were done with FreeSurfer 5.3.0 (Fischl, 2012). All visualized DICS results were smoothed via an iterative procedure utilizing sparse blurring matrices (Gramfort et al., 2014).

## 3 Results

### 3.1 Components

**FIGURE 1:**
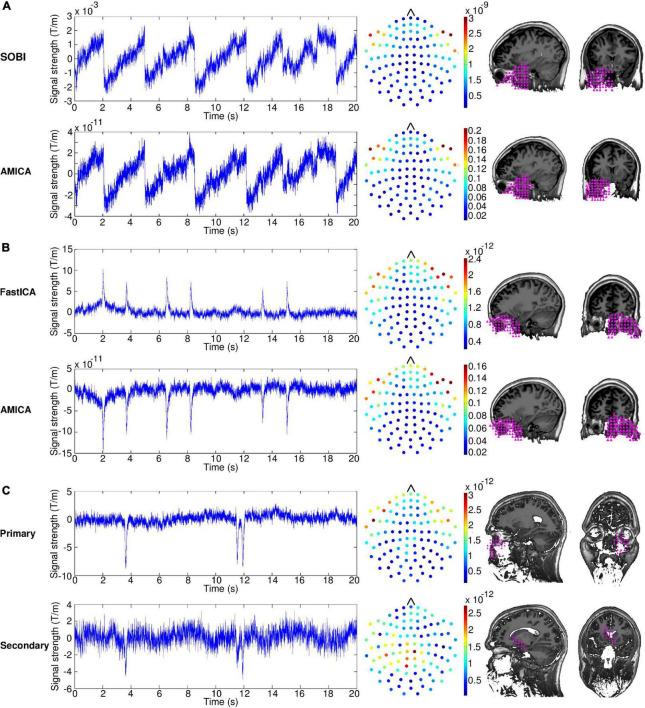
**(A)** Time series (left), sensor topographies (middle), and localization results (right) of the saccade component of a single subject for second-order blind identification (SOBI) and adaptive mixture ICA (AMICA). The dots in the sensor topography plots indicate the root mean squares of the mixing matrix weights of the two orthogonal gradiometers at a given location. The localization plots display sagittal and coronal slices at the level of the eye. The pink triangles indicate the locations that are the most probable source areas of the illustrated component. **(B)** Time series, sensor topographies and localization results of the blink component of a single subject for FastICA and AMICA. **(C)** Time series, sensor topographies and localization results of the primary and secondary blink component of a single subject extracted with FastICA.

Figure 1A shows examples of the time series, sensor topography and localization results of the saccade component extracted by SOBI and AMICA in one participant (Subject 4). In this case, the SOBI and AMICA components are almost identical in all these three characteristics. The time series exhibit the semiperiodic nature of reading-related saccades, with the signal rising steadily until it returns to the start position with the sharp descent that reflects the sweep to the beginning of the next line. At around 15 s, one can notice a sharp descent which is not as large as for the other sweeps and corresponds to a partial return sweep targeted to an already read part of the text on the same line. The scales of the components’ time series (*y*-axis) differ between the methods but this is compensated by a similar but inverse difference in the scales of the components’ weight matrices. This is a result of a difference in the way the methods scale the weight matrix and the signals. The sensor topographies show largest weights on the left- and rightmost frontal sensors, which are normally the sensors closest to the eyes. The weights decrease very steeply as the sensors’ distance from the eyes increases. The localization results indicate that the component most probably originated at the posterior wall of the left eye or behind it. This corresponds well to the proposal that a corneo-retinal potential is the origin of the saccadic signal. The component may also contain some activity from rectus muscles which rotate the eyes. The emphasis on the left eye in the localization is likely a result of the approximate nature of the localization method which stresses the most prominent origin of the component instead of yielding a more detailed spatial pattern.

For the blink component (Figure 1B, Subject 4), the results are very similar between the two alternative approaches, FastICA and AMICA. The time series contain several high-amplitude spikes, which correspond to the blink artifacts. The AMICA time series is inverted on the *y*-axis compared to the FastICA time series, but this is counterbalanced by inverted signs in the mixing matrices. The sensor topographies show major emphasis on the sensors located next to the eyes, with somewhat larger weights on the right side. The localization results are clustered very tightly on the right eye, reflecting the larger sensor weights on the right than left side.

The number of components required for extracting the artifacts in each subject are shown in Table 1. AMICA extracted the saccades into a single component in all subjects, whereas SOBI did the same in 12 subjects; in one subject, SOBI divided the saccades into two components with one component containing most of the saccade power and the other component only a minor part. Both approaches were also successful in extracting blinks, but the results were not as clear-cut as with saccades: AMICA extracted blinks into a single component in 11 subjects and into two components in two subjects, whereas FastICA extracted blinks into a single component in nine subjects, into two in three subjects and into three in one subject. For all subjects in whom AMICA required multiple components to extract the blinks, FastICA also required multiple components. Subject 7, for which FastICA extracted the blinks into three components and AMICA into two components, made unusually few blinks (see end of Subsection “2.1 Data” for the special data input scheme required in this case).

**TABLE 1:** Number of components required by SOBI-FastICA and AMICA to extract saccade and blink artifacts and the Pearson correlations between the components extracted by the different methods, per artifact type.

Subject no.	No. of saccade components (SOBI)	No. of saccade components (AMICA)	No. of blink components (FastICA)	No. of blink components (AMICA)	Saccade component correlation	Blink component correlation	No. of AMICA models, FastICA special data input (*)
1	2	1	1	1	–0.888	0.846	3
2	1	1	1	1	0.995	0.995	1
3	1	1	1	1	–0.967	0.962	1
4	1	1	1	1	0.990	–0.992	1
5	1	1	1	1	–0.920	–0.973	3
6	1	1	1	1	0.983	–0.991	3
7	1	1	3	2	–0.985	0.352	1, *
8	1	1	2	1	0.993	0.824	1
9	1	1	2	2	0.972	0.832	3
10	1	1	1	1	–0.995	–0.881	1
11	1	1	2	1	0.988	0.961	1
12	1	1	1	1	–0.955	0.902	1
13	1	1	1	1	–0.981	0.941	1

The negative sign in the correlation value indicates that the signs of the components were opposite but does not indicate any other dissimilarity. The last column contains the number of AMICA models estimated for the subject. Additionally, the subject requiring the special data input scheme with FastICA has been indicated with * in the last column.

As quantified by the Pearson correlations (Table 1), the artifact components extracted by the two alternative approaches were highly similar. Most of the correlations exceeded 0.9 and several 0.95. In many cases, however, the blink correlations were lower than the saccade correlations. In Subject 7, for whom many blink components were extracted by both pipelines, the blink components showed also a particularly low correlation. Most of the other lower correlations, the saccade correlation in Subject 1 and the blink correlations in Subjects 8 and 9, were also related to cases where one or both approaches required use of more than one component.

The case of Subject 8 is exemplified in Figure 1C, illustrating the time series, sensor topography and localization results for the primary and secondary blink components extracted by FastICA. The primary component is clearly a blink component resembling those presented in Figure 1B. The secondary component, however, is less salient and can be identified as a blink component only by the characteristic spike shape in the time series which appears concurrently with the spike shape of the primary component. The amplitude of the spike in the secondary component is markedly lower than that of the spike in the primary component indicating that it is a residual of the blink artifact not captured by the primary component. The largest weights in the sensor topography of the secondary component are on the sensors located over the temporal and parietal areas and the localization results imply that the component originated posterior to the eyes. This indicates that the secondary component is a mixture containing a substantial portion of other signals along with the blink residuals.

### 3.2 Effect on MEG data

**FIGURE 2:**
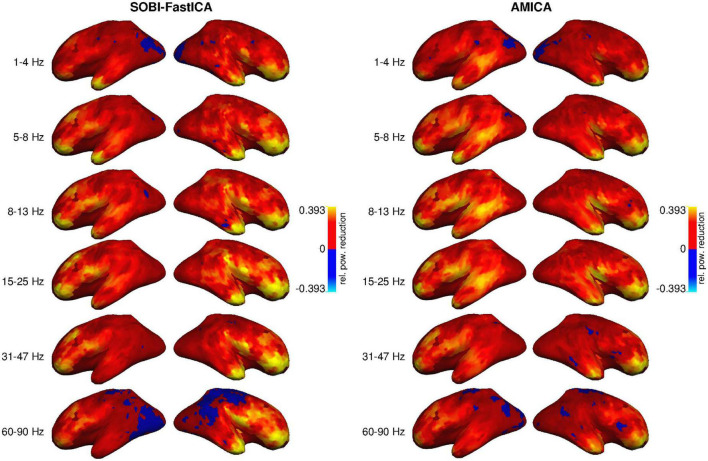
Reduction of signal power by SOBI-FastICA **(Left)** and AMICA **(Right)**, relative to the original uncleaned data, in six frequency bands (rows), averaged across subjects. More positive values indicate a larger reduction. In blue areas, the amount of reduced power was minimal and the DICS method erroneously estimated an increase in power (a negative reduction).

Figure 2 illustrates how much the removal of the artifact components using each of the two pipelines decreases signal power across the cortex in the six examined frequency bands. The effect appears to be strongest in the most anterior areas and the temporal poles, i.e., closest to the eyes, for all frequency bands and with both artifact removal methods. However, the removal also seems to affect, to a smaller degree, posterior parts of the frontal cortex and the temporal cortex, especially the inferior temporal cortex. The effect is minimal in the parietal cortex, the occipital cortex, and the most posterior part of the temporal cortex. The artifact removal operation impacts the whole range of frequencies from 1 to 90 Hz.

**FIGURE 3:**
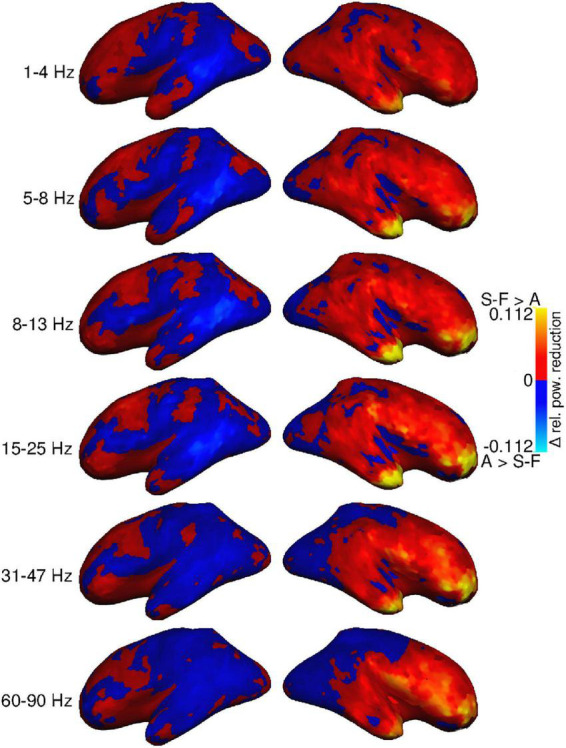
Group-level difference between power reduction by SOBI-FastICA and AMICA, relative to the power level of the AMICA pipeline. Red indicates areas where the SOBI-FastICA pipeline resulted in a larger reduction of signal power, whereas blue denotes areas where AMICA resulted in a larger reduction of signal power.

Differences in the size of the removal effect between the two alternative approaches were mostly subtle (Figure 3). The SOBI-FastICA combination seemed to reduce power more than AMICA in the right hemisphere, especially in the frontal areas, at all frequency bands. The difference was largest in the most anterior parts of the frontal cortex and the temporal poles, which are also the areas that the artifact removal generally affects the most. In the highest frequency bands, AMICA reduced power more in the posterior part of the right hemisphere. In the left hemisphere, there was more spatial variation regarding which pipeline reduced signal power the most with AMICA reducing power more in several areas throughout the hemisphere and especially in the temporal cortex. This spatial variation in the left hemisphere was present at all frequency bands.

**FIGURE 4:**
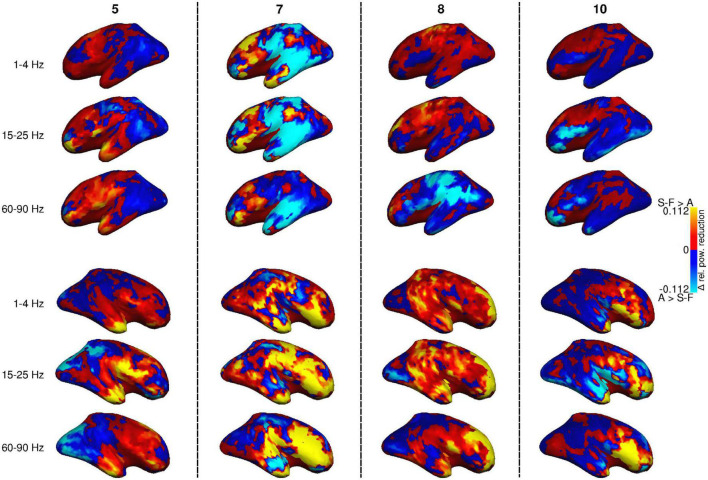
Difference between power reduction by SOBI-FastICA and AMICA in four individual subjects (no. 5, 7, 8, and 10; cf. Table 1) for frequency bands 1–4, 15–25, and 60–90 Hz, relative to the power level of the AMICA pipeline. Red indicates areas where the SOBI-FastICA pipeline resulted in a larger reduction of signal power, whereas blue denotes areas where AMICA resulted in a larger reduction of signal power.

There is, however, considerable subject-specific variation in the effects of the artifact removal pipelines. Figure 4 displays the power reduction difference maps for four individual participants (5, 7, 8, and 10 in Table 1) at three frequency bands (cf. Figures 2, 3 for the group-level view). Subjects 7 and 8 had more than one blink component in either the FastICA decomposition or both FastICA and AMICA decompositions, and subject 7 was the only participant with component correlation below 0.8 (see Table 1). The same general differences between the effects of the methods that are visible in the averaged maps of Figure 3 apply on the level of individual subjects: SOBI-FastICA generally reduces power more in anterior areas and dominates in the right hemisphere, whereas AMICA has a stronger effect in posterior areas and in the left hemisphere. Nevertheless, there can be large individual divergences from this template. As an especially notable example, in subject 10, AMICA reduced power more than SOBI-FastICA in several parts of the left and right frontal cortex and temporal poles, areas which in the grand-average plots in Figure 3 were dominated by the SOBI-FastICA pipeline.

**FIGURE 5:**
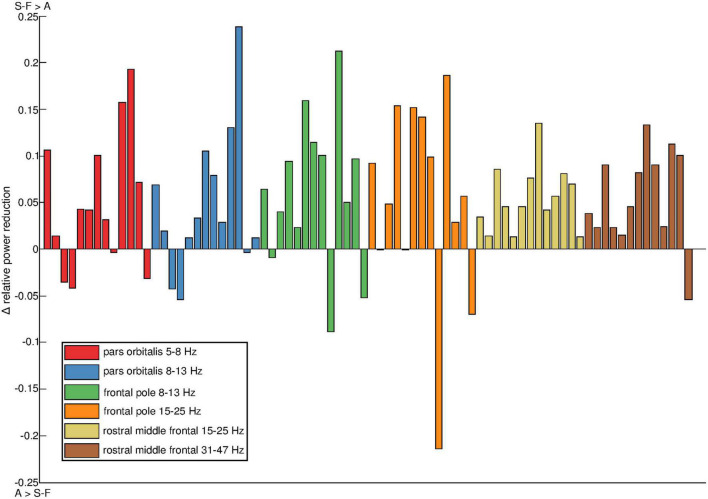
Individual variation of power reduction by SOBI-FastICA vs. AMICA in the six parcel and frequency band combinations for which the group-level difference between the methods exceeded 0.05. All such parcels were found in the right hemisphere. The results have been normalized by dividing them with the amount of power reduced by AMICA. Positive sign indicates that SOBI-FastICA reduced more power and negative sign that AMICA did. The colors denote the different parcel–frequency combinations. The colors were created with the linspacer function written by Lansey (2021) which uses colors derived mainly from ColorBrewer 2.0 (Brewer, 2021).

The intersubject variation in the effects of the artifact removal pipelines was examined further at parcel level. Figure 5 shows the distribution of power reduction differences in the individual participants, focusing on the subset of parcel-frequency band combinations for which the group-level difference in relative power reduction exceeded 0.05. All such parcel-frequency band combinations were concentrated to the right hemisphere. On average, SOBI-FastICA reduced power more than AMICA, but there was notable individual variation, even to the degree that in some subjects AMICA was markedly more efficient in reducing signal power (note, e.g., the large negative values in the frontal pole).

**FIGURE 6:**
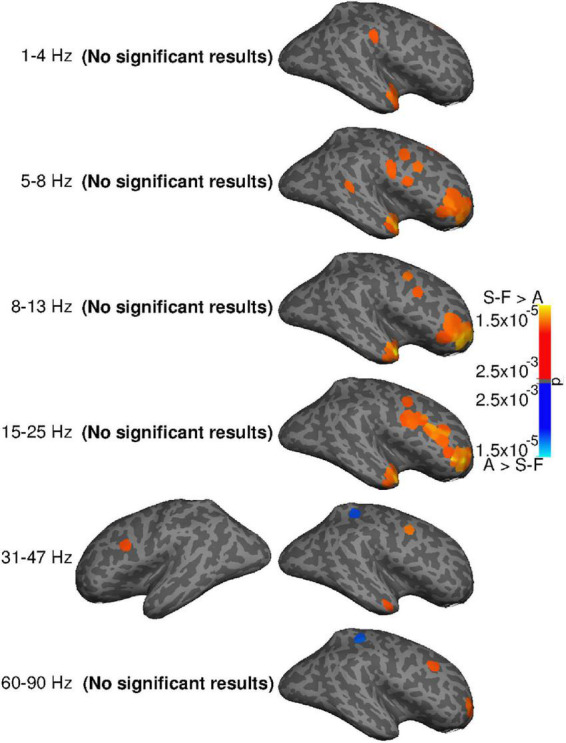
Systematic group-level differences (two-sided *t*-test, *p* ≤ 0.005, uncorrected) in power reduction by SOBI-FastICA and AMICA. *P*-values illustrated. The “(no significant results)” texts indicate that no significant results were found on the given hemisphere on the given frequency band. Orange indicates areas where the SOBI-FastICA pipeline reduced more power, and blue areas where AMICA reduced more power.

Statistical evaluation (Figure 6) confirmed systematic differences between the methods in the right frontal areas and temporal pole. In the frequency bands spanning 5–25 Hz, SOBI-FastICA reduced power more than AMICA, whereas AMICA reduced power more only at a single point in the right superior parietal area in the 31–47 and 60–90 Hz bands. In the left hemisphere, the only systematic difference was more power reduction with SOBI-FastICA than AMICA in the caudal middle frontal area at 31–47 Hz.

Table 2 summarizes group-level mean differences in power reduction between the pipelines for parcel-frequency band combinations containing points with systematic differences in power reduction (cf. Figure 6). The parcel-level differences are mostly well in line with the differences at single points, as SOBI-FastICA reduced power more than AMICA in the right frontal and temporal cortices, in multiple frequency bands. AMICA reduced power more in the right superior parietal parcel in the 31–47 and 60–90 Hz bands, but additionally also in the left precentral parcel in the 31–47 Hz band. The results for the left precentral parcel at 31–47 Hz are slightly anomalous: the parcel-frequency band combination was included in the table because SOBI-FastICA reduced power systematically more at one point at the anterior edge of the parcel, but on the level of the whole parcel, AMICA reduced power slightly more. The largest average power reduction differences in Table 2 are seen in the right rostral middle frontal, the superior temporal and the middle temporal cortices and the right pars triangularis. In the parcels where AMICA reduced power systematically more, the average differences are smaller than in the majority of the SOBI-FastICA-dominated parcels.

**TABLE 2:** Group-level mean differences in power reduction by SOBI-FastICA and AMICA for parcel-frequency band combinations containing points with systematic group-level differences (cf. Figure 6).

Parcel	1–4 Hz	5–8 Hz	8–13 Hz	15–25 Hz	31–47 Hz	60–90 Hz
Left precentral	–	–	–	–	−0.002	–
Right caudal middle frontal	–	0.014	0.017	0.026	0.024	0.026
Right postcentral	–	0.009	–	–	–	–
Right pars triangularis	–	0.039	0.047	–	–	–
Right supramarginal	0.011	–	–	–	–	–
Right banks superior temporal	–	0.021	–	–	–	–
Right middle temporal	–	–	0.032	0.033	0.026	–
Right rostral middle frontal	–	0.042	0.048	0.057	–	0.049
Right superior parietal	–	–	–	–	−0.009	−0.009
Right superior frontal	0.012	0.015	–	0.019	–	–
Right precentral	–	0.016	–	0.025	–	–
Right superior temporal	0.031	0.041	0.041	0.035	–	–

The results have been normalized by dividing them with the amount of power reduced by AMICA. Positive sign indicates that SOBI-FastICA reduced more power and negative sign that AMICA did. Dash denotes that no points with systematic differences were found.

## 4 Discussion

Our goal was to find an effective means of removing ocular artifacts from MEG data recorded during naturalistic reading of continuous text, while also testing whether it is feasible to extract the two major ocular artifact types, saccades and blinks, with one single method. We tested two alternative approaches, of which the first one used two different BSS methods, SOBI and FastICA that were deemed to be especially suitable for extracting either saccades or blinks, respectively. The second alternative utilized one purportedly powerful method, AMICA, for removing both artifact types. Both approaches proved to be effective, yielding components which were clearly artifactual based on their time series, the sensor-level spatial distribution, and source localization. In most cases, both approaches extracted the artifacts with just a single component per artifact type. Most of the components extracted from the same subject, but by a different method, yielded a Pearson correlation above 0.9, both for saccades and blinks. Almost all the cases where the correlation did not exceed 0.9 were those where either one or both pipelines yielded more than one artifact component. The grand-average DICS analysis of power reduction differences between the pipelines showed that the amount of signal power the pipelines reduced was on a comparable level in most cortical areas, except for the right frontal cortex, where SOBI-FastICA reduced systematically more power than AMICA across a wide range of frequencies.

### 4.1 Effects of different pipelines in artifact removal

Judged based on the number of components the pipelines required to extract the artifacts, saccades were more straightforward to identify than blinks. Saccades were extracted with a single component, except for one subject in whom SOBI-FastICA divided the artifact into two components. Blinks seemed to pose a slightly harder task with multiple components required in four subjects using SOBI-FastICA and in two subjects using AMICA. One possible explanation is that, in this data set, blinks occurred at a much lower rate than saccades, which may make it harder for the methods to treat them as a single artifact type and part of the same component. The number of subjects in whom multiple components were required was slightly higher for the SOBI-FastICA than AMICA pipeline. At least a partial reason for this can be that since saccades are removed first in the SOBI-FastICA pipeline, it is likely that parts of blink artifacts are already included in the saccade components and removed with them. Because of this, the remaining blink artifacts become fractured and thus more difficult to be perceived as a single component. AMICA, on the other hand, forms the components simultaneously, possibly allowing it to identify the artifacts as individual components.

The DICS estimation of power reduction showed that spatially the two artifact removal alternatives had similar effects on the data. Both affected signal power mainly in the frontal and temporal cortices, with mostly minor differences in the exact amount of signal removed from specific areas. However, in closer examination, the SOBI-FastICA pipeline reduced signal power systematically more than AMICA in the right frontal areas. As saccades in a continuous reading task are mostly directed rightwards, there could be a hemispheric imbalance in the saccade artifact and thus SOBI-FastICA pipeline might be more efficient than AMICA. However, because the saccade artifact is in practice a shift in the signal baseline which follows the direction of the gaze, the most important factor affecting the areal power distribution of the artifact is not the direction of the saccades, but the amount of time spent gazing in each direction. Since the text stimuli were presented at the center of the subjects’ field of view, saccades should not result in this kind of hemispheric imbalance. It is also possible that SOBI-FastICA removed more right-hemispheric cortical activity in addition to the artifacts than AMICA. Such activity could be for example related to eye movement control (Vernet et al., 2014) and partially coupled to the ocular artifacts. It is also possible that the synchronized movement of eyes during saccades that were present during a large portion of the recording induced high correlation levels within the data, thus leading to inaccurate beamforming estimates and the detected difference between the pipelines in the right frontal areas. However, as the placement of the eyes is symmetrical and as common beamformer weights were used in the estimation of cortical signal power, such inaccuracies are not very likely to cause the observed lateralized difference between SOBI-FastICA and AMICA pipelines.

As regards different MEG signal frequencies, the DICS estimations show that both artifact removal approaches had an effect across all frequency bands. This is not surprising, as the very non-sinusoidal step- or spike-like signal forms of the ocular artifacts mean that their power is spread over a wide frequency range, even though most of the ocular artifact power may lie in the range of 0–20 Hz (Keren et al., 2010). In the case of AMICA, power was indeed reduced most at lower frequencies from 0 to 25 Hz and notably less at the two highest frequency bands (31–47 and 60–90 Hz). The power reduction results for AMICA seem to reflect especially well the frequencies reported to contain most of the saccade power (4–20 Hz) (Keren et al., 2010), as the power reduction was weaker also at the lowest frequency band, which coincides with the frequencies of 0–4.5 Hz reported to contain most of the blink power (Keren et al., 2010). The primary ocular artifact frequencies were not reflected as clearly in the power reduction results for SOBI-FastICA.

These observations are based on results averaged over 13 subjects. However, it is also important to note there is considerable individual-level variation in the effects of the artifact removal methods, as the examination on the level of individual subjects shows. While the averaged results give a comprehensive overview of the general effects of the methods and their differences, the details of the results depend on the specific dataset, and a single subject’s data may thus not be affected in a way that is identical to the averaged results.

### 4.2 Methodological considerations

The main objective of this study was to compare the performances of a two-stage approach and a one-stage approach in ocular artifact removal. The performances were tested by using real measured data, which has the obvious disadvantage that it is impossible to fully know how much artifactual signal there is in the data, and what precisely is artifact and what is neural activity. Simulated data would give full control over these parameters. While simulations were our first choice, we eventually failed to generate data which would have been sufficiently similar to real data in order to be suitable for this type of performance testing. If the simulated data do not resemble real data, tests conducted on it may yield a far too optimistic view of the pipeline functionality and not give an accurate picture on how the tested methods perform in realistic artifact removal situations.

Suitable simulated data should meet essentially two criteria. Firstly, the signals constituting the data, especially the ones corresponding to artifacts, should closely resemble their real-life counterparts. Secondly, the simulated signals should mix in a sufficiently complex fashion, so that the task of identifying them is not too easy for the artifact removal methods. Our main approach to this problem was to randomly generate continuous signals and mix them based on a realistic source model. The ocular artifact signals were generated by using information on the different waveform characteristics and the appearance rate of the artifacts extracted from real measurements. The rest of the data consisted of a large number of signals built from randomly scrambled and distorted combinations of various basic waveforms and noise types. Nevertheless, we were not able to generate simulated data that would not have been excessively easy for the pipelines to unmix, except by overwhelming the component signals with an unrealistic amount of noise. Despite our failure, we encourage others to continue the development of such simulated data, which would be needed for definite quantitative testing and comparison of artifact removal methods.

The use of real measured data instead of simulated data also dictated our choice of metrics for the performance comparisons. As there is no precise knowledge of what is artifact and what is neural activity, it is difficult to define a measure that would yield quantitative information of the success of the artifact removal. This fundamental uncertainty undermines the usefulness of measuring, e.g., the amount of signal power removed at the times of ocular artifacts. Another potentially interesting metric, correlation between the extracted ocular artifact components and the EOG channels, has the same problems as using linear regression with EOG references as an ocular artifact removal method, namely that EOG channels contain also neural activity, and that EOG measures electrical activity whereas MEG measures magnetic activity.

In the present study, we chose to focus on SOBI, FastICA and AMICA in removing ocular artifacts from the MEG data. Of these, the choice of AMICA was unambiguous due to the high performance level and the flexibility of the method. As regards SOBI, while its operational principles are very well-suited for the removal of saccades from continuous reading data, similar but potentially more effective approaches have been proposed (Congedo et al., 2008). However, SOBI was chosen as it is arguably a more widely known method. Also, for FastICA, a few noteworthy alternatives may exist. Some studies comparing different artifact removal methods indicate that, of the more well-known ICA methods, JADE, Infomax or Extended Infomax, of course AMICA, or even some non-ICA methods (see e.g., Romero et al., 2008; Delorme et al., 2012) might perform better than FastICA in selected scenarios. Both of the methods in the SOBI-FastICA pipeline were deemed necessary in separate tests where both the ability of SOBI to extract blinks and the ability of FastICA to extract saccades were observed to be insufficient. The processing with SOBI and FastICA was performed in this particular order, because we observed that removing blinks first may prevent SOBI from finding saccades very effectively.

Preprocessing methods are also an important part of artifact removal pipelines. We kept the preprocessing part of our pipelines minimal in order to concentrate on the effects that the SOBI-FastICA pipeline and AMICA have on the data. One notable preprocessing step that was left out of the study is high-pass filtering, which has been shown to be beneficial in conjunction with ICA (e.g., Winkler et al., 2015). In our own limited tests with these pipelines and data, high-pass filtering resulted in saccade components becoming more mixed with other activity, thus effectively decreasing the performance of the pipelines. This performance reduction, which seems to contradict the usual observations of the benefits of high-pass filtering, may result from the use of MEG instead of EEG. EEG measurements often suffer from various types of low-frequency drifts due to, e.g., electrode movement that do not appear similarly in MEG measurements. As a result, filtering these drifts out from EEG data facilitates the extraction of ocular artifacts from EEG data, whereas in MEG this may in fact impede the identification of ocular artifacts by reducing the amount of information available on them.

The present results are influenced also by our criteria of labeling components as ocular artifacts, which we based on visual inspection of the components’ time series and sensor topographies as well as source localization. This choice was especially inspired by Jung et al. (2001), who used similar criteria for removing blink artifacts from event-related potentials. The analyses and estimation of sensor topographies was based only on gradiometers as they more readily allow the identification of artifactual spatial configurations than magnetometers and as the choice of the sensor type has only a small impact on source-level estimates of neural activity (Garcés et al., 2017). Multiple other indicators for detecting artifactual components have been suggested especially for automatic artifact removal (Delorme et al., 2001; Barbati et al., 2004; Escudero et al., 2007; Nolan et al., 2010; Mognon et al., 2011; Winkler et al., 2011; Plöchl et al., 2012; Chaumon et al., 2015), but we did not find in the literature alternative or additional criteria that would have yielded further useful information in identifying artifacts with certainty in manual inspection. Resorting to automatic detection was not an option here, since it was of utmost importance that the components labeled as artifacts could be verified to contain artifactual activity, and it was only possible to do this manually. Concurrently recorded eye tracker data was not explicitly used here in artifact identification (apart from guiding to artifacts in time series inspection), as eye-tracker-based criteria did not seem to offer any true benefit beyond the criteria now used.

As an additional criterion for detecting artifact components, left out from this study as redundant but potentially useful in other studies, we can recommend calculating the Pearson correlation between the components and the EOG channels (e.g., Joyce et al., 2004; Nolan et al., 2010; Chaumon et al., 2015). High correlation with the horizontal EOG channel may suggest a saccade component and a high correlation with the vertical EOG channel a blink component. If eye tracker data is available, it may be used to increase the accuracy of this criterion especially for blinks by performing the calculation only on segments which are known to contain artifacts. We point out that even though correlation with EOG channels does not work as an absolute measure of the quality of the artifact removal process, it is still suitable for locating possible artifactual activity from components.

A final methodological choice which may have impacted our results is the way we have divided the data when feeding it to the pipelines. We chose to process all the data from a single subject at once (both reading task and scanning task) in order to ensure that the effects of artifact removal are uniform across the data. This kind of uniformity is desirable because the data from the two tasks would be contrasted when analyzed from the perspective of reading research and any differences between the tasks resulting from artifact removal may confound the analysis. A problem which may arise from the use of such long, partially concatenated data is the lack of stationarity, meaning that the properties of the signals differ significantly at different time points in the data. For ocular artifacts, this could mean significant changes in their amplitudes, appearance rate, some other statistical properties or spatial topography, for example as a result of small head movement. However, in the study of continuous data it is implicitly assumed that the signals are stationary, i.e., it is possible to study the phenomenon of interest by using the entire recording. This assumption of stationarity may similarly be extended to the removal of artifacts. Additionally, minor changes in the properties of the artifacts are not expected to hinder their extraction substantially and may simply lead to an increase in the amount of other signals mixing into the artifact components. If uniformity of artifact removal across different experimental conditions is not required for the study, the data could be processed in shorter pieces. For example Jung et al. (2000) have suggested using epochs of only 10 s in duration.

### 4.3 Usability of the methods

In addition to the effectiveness of the method, the usability of the method may also influence the choice of the artifact removal tool. SOBI is available in Matlab at least as a part of the EEGLAB and FieldTrip toolboxes and as solitary implementations. In Python, SOBI is available both as user written implementations and as a part of the Shogun toolbox (Sonnenburg et al., 2010). FastICA is also readily available and can be applied in Matlab through the official FastICA toolbox. A Python implementation of FastICA is found at least from the MNE (Gramfort et al., 2013) and scikit-learn (Pedregosa et al., 2012) toolboxes.

AMICA is available in Matlab as a solitary implementation as well as a plug-in in the EEGLAB toolbox. According to our knowledge, no Python implementations are currently in distribution. Setting up a working AMICA pipeline (without EEGLAB) proved somewhat time-consuming. A large part of the code in the AMICA implementations is wrapped into a binary and therefore not easily modifiable. AMICA has a large set of parameters and a somewhat complex output structure, with a rather limited set of instructions. However, the default values of the parameters seemed to be well set for basic use, with only the number of models requiring adjustment in some cases.

### 4.4 Conclusion

We conclude that ocular artifact removal on naturalistic MEG reading data can be performed effectively with both the combination of SOBI and FastICA as well as a single overall powerful method in the form of AMICA. Our cautious recommendation is to use AMICA because the use of a single method instead of two reduces the uncertainty inherent in ocular artifact removal as it is easier to inspect all the different components when they come from only one method. The nature of AMICA as an overall powerful method means also that it can be used more readily for removing all desired types of artifacts in situations where the need for artifact removal is not limited to ocular artifacts. However, if the use of AMICA is not feasible, we find the SOBI-FastICA pipeline to be a highly recommendable alternative for removing artifacts from MEG data on naturalistic reading of continuous texts.

## Data availability statement

The datasets presented in this article are not readily available because of the ethical permission and national privacy regulations at the time of the study. The data that support the findings of this study are available from the corresponding author with permission of the Aalto Research Ethics Committee. Requests to access the datasets should be directed to SM, sasu.makela@aalto.fi.

## Ethics statement

The studies involving human participants were reviewed and approved by Helsinki and Uusimaa Ethics Committee. The participants provided their written informed consent to participate in this study.

## Author contributions

SM: conceptualization, methodology, software, investigation, writing – original draft, and writing – review and editing. JK: conceptualization, methodology, writing – review and editing, and supervision. RS: conceptualization, writing – review and editing, supervision, and funding acquisition. All authors contributed to the article and approved the submitted version.

## Footnotes

1
https://github.com/fieldtrip/fieldtrip/blob/master/external/eeglab/sobi.m

2
https://research.ics.aalto.fi/ica/fastica/

3
https://sccn.ucsd.edu/∼jason/amica_web.html
