# Supplementary material for: Removing ocular artifacts from magnetoencephalographic data on naturalistic reading of continuous texts
Source: Front Neurosci. 2022 Dec 22;16:974162. doi: 10.3389/fnins.2022.974162 (PMC9815455; doi:10.3389/fnins.2022.974162)
Supplement: Supplementary file 1 [file Data_Sheet_1.pdf]

Appendix

1 SOBI

We present here the basics of the SOBI algorithm as outlined in Belouchrani et al. (Belouchrani et al., 1997). Details of the whitening procedure used in this version of the method are not explained here but can be found in Belouchrani & Cichocki (Belouchrani & Cichocki, 2000). The method consists of four main steps: 1. centering and whitening of the data $\boldsymbol{x}(t)$, 2. forming sample covariance matrices $\boldsymbol{R}_x(\tau_j), \mathrm{j} = 1 \ldots \mathrm{K}$ of the whitened signals for a fixed set of time lags τ_j, 3. finding a unitary matrix $\boldsymbol{U}$, which is a joint diagonalizer for the sample covariance matrices $\boldsymbol{R}_x(\tau_j)$, and 4. calculating the mixing matrix $\boldsymbol{A}$ and source signals $\boldsymbol{s}(t)$ with $\boldsymbol{U}$. The aim of centering is to force all variables in the data to have a mean value of zero, whereas whitening alters the variables such that they are uncorrelated and their variance is one. The purpose of these preprocessing steps, which also appear in many other BSS methods, is to simplify the operation of the rest of the method. In order to calculate the sample covariance matrices $\boldsymbol{R}_x(\tau_j)$, the time lags τ_j must be set. Since $\boldsymbol{R}_x(\tau_j)$ effectively form the data which the demixing is based on, it is possible to try and guide the method to concentrate on certain signals by selecting τ_j that emphasize some desired parts of the measured data.

After the $\boldsymbol{R}_x(\tau_j)$ have been calculated, their diagonalizer $\boldsymbol{U}$ can be estimated. This demands the formulation of the ”off” function. The ”off” of an $n\mathrm{x}n$ matrix M is the sum of all off-diagonal elements of $\boldsymbol{M}$**,** formally expressed as:

$$off(\boldsymbol{M}) = \sum_{i,j=1,i\neq j}^{n} \left|M_{ij}\right|^2. \quad (1)$$

The ”off” can be used to define a diagonalizer matrix in the way that a unitary matrix $\boldsymbol{V}$ is a diagonalizer for $\boldsymbol{M}$, if $off(\boldsymbol{V}^T\boldsymbol{M}\boldsymbol{V}) = 0$. Since diagonalizing several matrices with a single matrix may not always be possible, SOBI tries to find the best possible joint diagonalizer $\boldsymbol{U}$ for the $\boldsymbol{R}_x(\tau_j)$ by finding $\boldsymbol{U}$**,** which minimizes the criterion $C(\boldsymbol{U}, \mathcal{R})$:

$$\boldsymbol{C}(\boldsymbol{U}, \boldsymbol{\mathcal{R}}) = \sum_{j=1}^{K} off(\boldsymbol{U}\boldsymbol{R}_x(\tau_j)\boldsymbol{U}), \quad (2)$$

where $\mathcal{R}$ is the set of matrices $\mathcal{R} = \{\boldsymbol{R}_x(\tau_1), \ldots, \boldsymbol{R}_x(\tau_K)\}$. Details of the algorithm for minimizing $C(\boldsymbol{U}, \mathcal{R})$ can be found in Belouchrani et al. (Belouchrani et al., 1997). Finally, after $\boldsymbol{U}$ has been estimated the source signals $\boldsymbol{s}(t)$ and the mixing matrix $\boldsymbol{A}$ can be calculated as

$$\boldsymbol{s}(t) = \boldsymbol{U}^T\boldsymbol{Z}\boldsymbol{x}(t), \quad (3)$$

where $\boldsymbol{Z}$ is the matrix for whitening the data, and

$$\boldsymbol{A} = \boldsymbol{W}^{-1}\boldsymbol{U}. \quad (4)$$

2 FastICA

Since calculating the mutual information according to its formal traditional definition would also require knowledge of the signals’ probability distributions, FastICA takes advantage of an alternative way of expressing the mutual information I, applicable for uncorrelated variables (Comon, 1994):

$$I(y_1, y_2, \dots, y_n) = J(\boldsymbol{y}) - \sum_{i=1}^{n} J(y_i) \tag{5}$$

$$y_i = \boldsymbol{w}_i^T \boldsymbol{x}, \tag{6}$$

where the variables y_i are calculated with the current linear transformation and form the vector $\boldsymbol{y} = (y_1, y_2, \dots, y_n)$, $J(y_i)$ denotes the negentropy of these variables y_i and $J(\boldsymbol{y})$ the joint negentropy of the variables y_i, the negentropy of the vector $\boldsymbol{y}$. Negentropy can be seen both as a normalized version of differential entropy, the measure of the expected value of information in one observation of a variable, and a measure of the nongaussianity of the variable (Comon, 1994).

According to Equation 5, mutual information can be minimized by aiming for a linear transformation which maximizes the negentropies $J(y_i)$ of the variables y_i. This task requires a means of calculating the negentropies $J(y_i)$, which is done in FastICA by using the approximation

$$J(y_i) = J_G(\boldsymbol{w}_i) \approx [E(G(\boldsymbol{w}_i \boldsymbol{x}) - E(G(v)))]^2, \tag{7}$$

where $E()$ denotes the expectation, $G()$ is an arbitrary contrast function (to be explained below) and v is a Gaussian variable of zero mean and unit variance. Equation 7 shows also more explicitly that the variables y_i can only be altered by altering the weights $\boldsymbol{w}_i$ since the observed data $\boldsymbol{x}$ is fixed (see Equation 6). FastICA includes several options for the contrast function $G()$ with varying properties. Of these, we used the function

$$G(u) = \frac{-1}{a} \exp(\frac{-au^2}{2}), \tag{8}$$

chosen for its robustness (Hyvärinen, 1999). exp() denotes the exponetial function and a is an adjustable constant for which we used the default value 1.

Finally, the core algorithm of FastICA takes the form of the following optimization problem:

$$\textit{maximize} \sum_{i=1}^{n} J_G(\boldsymbol{w}_i)$$

$$\textit{under constraint } E\left((\boldsymbol{w}_k^T \boldsymbol{x})(\boldsymbol{w}_j^T \boldsymbol{x})\right) = \delta_{jk}.$$

Here δ_{jk} is the Kronecker delta. The maximization is performed by using a variation of the Newton method, the details of which can be found in Hyvärinen (Hyvärinen, 1999). The constraint requires that the calculated components of the transformation are orthogonal. For the orthogonalization FastICA offers two alternatives, of which the sequential option was used in this study. Finally, after the maximization has been performed, the $\boldsymbol{w}_i$ form the rows of the demixing matrix W and the vector $\boldsymbol{y}$ becomes the source signal $\boldsymbol{s}$.

3 AMICA

The following description of AMICA is mostly based on the one provided by Hsu et al. (Hsu et al., 2018). As a result of AMICA's option for multiple models, the basis equation of BSS (see Equation 1 in the article) takes the following form in AMICA:

$$x(t) = x_h(t) = \boldsymbol{A}_h \boldsymbol{s}_h + \boldsymbol{c}_h, h = 1, \dots, H, \tag{9}$$

where $\boldsymbol{c}_h$ is a bias term, the subscript h denotes the index of the model and H he number of different models. The likelihood of the data given the models is expressed as:

$$p(\boldsymbol{X}|\Theta) = \prod_{t=1}^{T} \sum_{h=1}^{H} p(x(t)|C_{h,}\Theta_h) \cdot p(C_h), \tag{10}$$

where $\Theta = (\theta_1, \ldots, \theta_H)$ contains the parameters of the models and $p(C_h) = \gamma_h$ denotes the probability of the model h being active. The activity probabilities γ_h of the different models sum to 1. Combining Equations 9 & 10, the likelihood of the observed signals $\boldsymbol{x}(t)$ given the model can be expressed in terms of the demixing matrices $\boldsymbol{W}_h$ of the different models and the probabilities of the ICA components $s_{hi}(t)$ (index i is the index of the component in the given model) as:

$$p(x(t)|C_h, \Theta_h) = |\det \boldsymbol{W}_h| \cdot \prod_{i=1}^{N} p(s_{hi})(t), \tag{11}$$

where $\det \boldsymbol{W}_h$ denotes the determinant of the demixing matrix $\boldsymbol{W}_h$, $p(s_{hi}(t))$ the value of the probability density function of the component $s_{hi}(t)$ at time t and N the number of ICA components. The probability density function of each component $p(s_{hi}(t))$ is approximated as a mixture of generalized Gaussian distributions $q(s)$:

$$p\big(s_{hi}(t)\big) = \sum_{j=1}^{M} \alpha_{hij} \cdot q(s_{hi}(t); \rho_{hij}, \mu_{hij}, \beta_{hij}), \tag{12}$$

where α_{hij} is the weight of each generalized Gaussian distribution, subscript j denotes the index of the distribution and M the number of different distributions. The generalized Gaussian distribution can be determined by its shape ρ, location μ and scale β parameters and expressed as:

$$q(s; \rho, \mu, \beta) = \frac{\rho}{2\beta \cdot \Gamma(\frac{1}{\rho})} \exp\left(-\left|\frac{s-\mu}{\beta}\right|^{\rho}\right), \tag{13}$$

where Γ denotes the gamma function. Accordingly, the model applied in AMICA consists of the parameters $\boldsymbol{W}_h$, $\boldsymbol{c}_h$, γ_h, α_{hij}, β_{hij}, ρ_{hij}, μ_{hij}. These parameters are estimated by using the expectation-maximization algorithm in conjunction with a fast-converging variation of the Newton method derived by Palmer et al. (Palmer et al., 2008).

4 References

Belouchrani, A., Abed-Meraim, K., Cardoso, J.-F.. & Moulines, E. (1997). A blind source separation technique using second-order statistics. IEEE Transactions on Signal Processing, 45(2), 434-444. https://doi.org/10.1109/78.554307

Belouchrani, A., & Cichocki, A. (2000). Robust whitening procedure in blind source separation context. Electronics Letters, 36(24), 2050-2051. https://doi.org/10.1049/el:20001436

Comon, P. (1994). Independent component analysis, a new concept? Signal Processing, 36(3), 287-314. https://doi.org/10.1016/0165-1684(94)90029-9

Hsu, S. H., Pion-Tonachini, L., Palmer, J., Miyakoshi, M., Makeig, S., & Jung, T. P. (2018). Modeling brain dynamic state changes with adaptive mixture independent component analysis. NeuroImage, 183, 47-61. https://doi.org/10.1016/j.neuroimage.2018.08.001

Hyvärinen, A. (1999). Fast and robust fixed-point algorithms for independent component analysis. IEEE Transactions on Neural Networks, 10(3), 626-634. https://doi.org/10.1109/72.761722
